# A Prospective Analysis of the Retinopathy of Prematurity Correlated with the Inflammatory Status of the Extremely Premature and Very Premature Neonates

**DOI:** 10.3390/diagnostics13122105

**Published:** 2023-06-18

**Authors:** Claudia Ioana Borțea, Ileana Enatescu, Mirabela Dima, Manuela Pantea, Emil Radu Iacob, Catalin Dumitru, Alin Popescu, Florina Stoica, Rodica Elena Heredea, Daniela Iacob

**Affiliations:** 1 Department of Neonatology, “Victor Babes” University of Medicine and Pharmacy Timisoara, Eftimie Murgu Square 2, 300041 Timisoara, Romania; bortea.ioana@umft.ro (C.I.B.); enatescu.ileana@umft.ro (I.E.); dima_mirabela@yahoo.com (M.D.); manu.pantea@gmail.com (M.P.); iacob.daniela@umft.ro (D.I.); 2 Doctoral School, “Victor Babes” University of Medicine and Pharmacy Timisoara, Eftimie Murgu Square 2, 300041 Timisoara, Romania; 3 Department of Pediatric Surgery, “Victor Babes” University of Medicine and Pharmacy Timisoara, Eftimie Murgu Square 2, 300041 Timisoara, Romania; radueiacob@umft.ro; 4 Department of Obstetrics and Gynecology, “Victor Babes” University of Medicine and Pharmacy Timisoara, Eftimie Murgu Square 2, 300041 Timisoara, Romania; popescu.alin@umft.ro; 5 Department of Ophthalmology, “Victor Babes” University of Medicine and Pharmacy Timisoara, Eftimie Murgu Square 2, 300041 Timisoara, Romania; florinastoica@gmail.com; 6 Department of Pathology, “Louis Turcanu” Children’s Clinical Emergency Hospital, 300041 Timisoara, Romania; elena-rodica.heredea@umft.ro; 7 Department of Clinical Practical Skills, “Victor Babes” University of Medicine and Pharmacy Timisoara, Eftimie Murgu Square 2, 300041 Timisoara, Romania

**Keywords:** prematurity, preterm birth, retinopathy, inflammatory markers, inflammation

## Abstract

Retinopathy of Prematurity (ROP) is a major cause of blindness in premature infants. This study aimed to evaluate the association between inflammatory markers and ROP development in extremely premature and very premature neonates and identify potential inflammatory biomarkers for ROP risk prediction. This prospective study was conducted from January 2021 to January 2023 in two clinical hospitals associated with the “Victor Babes” University of Medicine and Pharmacy Timisoara. The study population comprised neonates with a gestational age of less than 32 weeks. Various inflammatory markers, including total white blood cell count, polymorphonuclear leukocytes, C-reactive protein, interleukin-6, and lactate dehydrogenase, were analyzed from blood samples collected at birth and three days postnatally. ROP was diagnosed and classified following the International Classification of Retinopathy of Prematurity. The study included 48 neonates, 12 Extremely Premature Infants (EPI), and 36 Very Premature Infants (VPI). The EPI group had significantly higher mean interleukin-6 and lactate dehydrogenase levels at birth and three days postnatally than the VPI group. C-reactive protein levels at three days were significantly higher in the VPI group. Umbilical cord inflammation and ROP severity were found to have a statistically significant positive correlation. Half of the EPIs had moderate to severe ROP, significantly more than in the VPI group. The duration of oxygen supplementation, mechanical ventilation, Continuous Positive Airway Pressure (CPAP), gestational age less than 28 weeks, and umbilical cord inflammation at or above stage 3 were significant risk factors for developing ROP stage 2 or above. Elevated CRP and IL-6 were also significantly associated with an increased risk of developing ROP stage 2 or above, highlighting their potential as biomarkers for ROP risk prediction. This study suggests a significant association between inflammatory markers and ROP development in extremely premature and very premature neonates. These findings could contribute to the identification of potential inflammatory biomarkers for ROP risk prediction, improving early diagnosis and intervention strategies for this condition.

## 1. Introduction

Retinopathy of Prematurity (ROP) is a potentially blinding eye disorder [1,2] that primarily affects premature infants, particularly those born extremely premature, before 28 weeks gestation, or very premature, between 28 to 32 weeks of gestation [3,4]. It is characterized by the abnormal growth of blood vessels in the retina, which can lead to retinal detachment and, eventually, blindness if left untreated [5,6,7]. The prevalence of ROP has increased significantly in recent years due to the improved survival rates of premature infants, making it an important public health concern [8].

The pathogenesis of ROP is multifactorial, involving a complex interplay of various factors such as oxygen levels, vascular growth factors, and inflammatory mediators [9,10]. Inflammation has been suggested to play a crucial role in developing ROP, with studies showing elevated levels of pro-inflammatory cytokines in the vitreous humor and serum of infants with ROP [11,12,13]. However, the specific role of inflammation in the pathogenesis of ROP, and its association with the severity of the disease, remains unclear.

Extremely premature and very premature neonates are known to be at a higher risk of developing ROP due to their underdeveloped retinal vasculature and their increased susceptibility to oxidative stress and inflammation [14]. In these infants, the immature immune system and exposure to various stressors, such as infections and mechanical ventilation, can result in a heightened inflammatory response, further contributing to the development and progression of ROP [15,16,17].

Several studies have investigated the association between inflammatory markers and the risk of ROP, focusing on specific cytokines, such as interleukin (IL)-6, IL-8, tumor necrosis factor-alpha (TNF-α), and insulin-like growth factor (IGF) [18,19,20]. However, the results have been inconsistent, with some studies showing a significant association between elevated cytokine levels and ROP, while others have not found any correlation [21,22]. Furthermore, most of these studies have been retrospective, with a limited ability to establish a causal relationship between inflammation and ROP.

Given the inconsistencies in the literature and the importance of understanding the role of inflammation in ROP, there is a need for prospective, longitudinal studies that can assess the inflammatory status of extremely premature and very premature neonates and determine its association with the development and progression of ROP. Therefore, the present study aims to conduct a prospective assessment of the development of ROP and its relationship with the inflammatory status of extremely premature and very premature neonates. By longitudinally evaluating the levels of various inflammatory markers, we hope to better understand the role of inflammation in the pathogenesis of ROP and identify potential biomarkers for predicting the risk of ROP in these vulnerable infants.

## 2. Materials and Methods

### 2.1. Study Design and Ethical Considerations

The specific hypotheses of this study were that (1) higher levels of inflammatory markers will be associated with an increased risk of ROP development and progression, and (2) extremely premature and very premature neonates with ROP will exhibit a distinct inflammatory profile compared to those without ROP. The study objectives are to evaluate the association between the levels of various inflammatory markers and the development and progression of ROP in extremely premature and very premature neonates and to identify potential inflammatory biomarkers for ROP risk prediction. 

This study was a prospective research project conducted over two years, from January 2021 to January 2023. The study setting involved the Maternity Hospital and the neonatal intensive care unit (NICU) of the “Pius Brinzeu” County Emergency Clinical Hospital Timisoara and the “Louis Turcanu” Children’s Emergency Clinical Hospital Timisoara, both associated with the “Victor Babes” University of Medicine and Pharmacy Timisoara (UMFVBT). Ethical approval was obtained from the UMFVBT’s local ethics committee, ensuring strict adherence to the International Conference on Harmonization’s guidelines on human research (approval number 3 from 8 January 2021).

### 2.2. Inclusion and Exclusion Criteria

The study population comprised premature neonates, specifically those born vaginally with a gestational age of less than 32 weeks (up to 31 weeks and six days) and those admitted to the NICU. Premature labor was characterized by continuous uterine contractions that occurred at least twice every 10 min, leading to birth before the completion of 37 full weeks of gestation. The inclusion criteria for the study did not place any restrictions based on birth weight. However, the lower limit for fetal viability was defined as a gestational age of 22 weeks [23]. Neonates who fell into the category of extreme prematurity were those born earlier than 28 weeks, while very premature neonates were defined as those born between 28 and 32 weeks of gestation [24].

Several exclusion criteria were also set for the study: neonates born after 32 weeks of gestation, neonates delivered via Cesarean section, and neonates with a gestational age of 22 weeks or less, which was considered below the threshold of viability. Other exclusion criteria incorporated neonates with congenital anomalies or a critical illness that might impact the inflammatory response. Examples of such conditions include congenital heart disease, congenital infections, or chromosomal abnormalities [25]. Neonates whose mothers had experienced infections or inflammatory conditions during pregnancy were also excluded, as these conditions could affect the neonate’s inflammatory status [26]. Additionally, neonates who did not survive the neonatal period were excluded, as the progression of ROP could not be assessed in these infants. Lastly, parents who did not consent to data collection and participation in research studies, or those with psychiatric diseases that impaired the ability to consent, were also excluded from this study [27,28]. These criteria ensured a homogeneous study population, allowing findings to be attributed to the variables under investigation. 

### 2.3. Laboratory and Histopathology Methods

Umbilical cord histochemical analysis was conducted on a 10 cm segment from the distal third of the umbilical cord collected at birth. The sample was preserved in formalin for transport to the pathology laboratory, fixed in paraffin, and examined microscopically by the same pathologist. Blood samples were collected at birth and three days postnatally for the determination of total white blood cell (WBC) count, polymorphonuclear leukocytes (PMN), C-reactive protein (CRP), interleukin-6 (IL-6), and lactate dehydrogenase (LDH). The blood was placed in ethylenediaminetetraacetic acid (EDTA) tubes and stored at +4 degrees Celsius until analysis. The extent of umbilical cord inflammation was determined based on existing guidelines, with a four-stage grading system employed based on the extent of neutrophil infiltration into the umbilical vascular walls [29]. The normal range for the absolute neutrophil count in neonates was between 5000 and 20,000 cells/μL, while the PMN ratio was between 40 and 60% [30].

ROP was diagnosed and classified following the International Classification of Retinopathy of Prematurity (ICROP) [31]. The diagnostic examinations for ROP were performed by an experienced ophthalmologist using indirect ophthalmoscopy (Vantage Plus, Keeler, Windsor, UK). The initial ophthalmoscopic examination was performed 1 h after the first feeding and weekly until four weeks after birth. Ophthalmoscopy was performed in mydriasis with cyclopentolate 0.5%, tropicamide 0.5%, or phenylephrine 2.5%. The severity of ROP was categorized into five stages, ranging from mild (stage 1) to severe (stage 5). Plus, ROP disease, characterized by abnormal dilation and tortuosity of the retinal blood vessels, was also noted if present, as it indicates a more severe disease.

### 2.4. Study Variables

The primary study variables included the levels of various inflammatory markers (PMN ratio, C-reactive protein, IL-6, and LDH), the degree of umbilical cord inflammation, and the development and progression of ROP. Other variables included the number of erythrocyte transfusions, days with oxygen supplementation, mechanical ventilation, the proportion of patients on CPAP, surfactant administration, and mortality. ROP diagnostic examinations were performed by an experienced ophthalmologist using indirect ophthalmoscopy, with immediate therapeutic interventions performed for infants that developed Stage 3 ROP.

### 2.5. Statistical Analysis

A convenience sample method was employed, with 48 subjects included in the analysis. Data normality was assessed using the Kolmogorov–Smirnov test. Mean and standard deviation were used to describe normally distributed data, while the Student’s *t*-test was used to compare means between the two groups. Proportions were presented as *n* (%), with the Chi-square test or the Fisher’s exact test used for comparisons. Correlations were described using Spearman’s and Pearson’s correlation coefficients. A multivariate regression analysis was used to identify the risk factors for ROP. Statistical significance was set at a *p*-value of less than 0.05.

## 3. Results

### Study Demographics

The study included a total of 48 neonates, categorized into Extremely Premature Infants (EPI) with a gestational age of less than 28 weeks (*n* = 12) and Very Premature Infants (VPI) with a gestational age between 28 and 32 weeks (*n =* 36). Demographic and clinical characteristics were compared between the two groups. The mean birth weight of the total population was 1291.8 g, with a standard deviation of 405.8. A significant difference in mean birth weight was found between the EPI and VPI groups (864.5 ± 231.4 g vs. 1392.7 ± 364.2 g, respectively), with a *p*-value of less than 0.001, indicating a statistically significant difference. When analyzed by weight range, a higher proportion of EPIs fell within the 500–1000 g range (66.7%) compared to the VPIs (19.4%), while all the neonates in the 1500–2000 g range were from the VPI group. However, the weight range comparison yielded a *p*-value of 0.004, which suggests a significant difference between the two groups.

In terms of gender, the study found no significant difference between EPIs and VPIs, with approximately equal proportions of males and females in each group (*p* = 0.738), as presented in Table 1. The gestational age distribution showed that all the neonates in the 24–27 weeks of gestation range were in the EPI group, while the VPI group consisted of all neonates in the 28–31 weeks of gestation range. This distribution is consistent with the definitions of EPI (less than 28 weeks of gestation) and VPI (28–32 weeks of gestation).

The two groups had a significant difference in the mean level of Interleukin 6 (IL-6). The EPI group had a markedly higher mean IL-6 level (638.2 ± 122.7 pg/mL) than the VPI group (151.1 ± 26.7 pg/mL), with a *p*-value less than 0.001, indicating a statistically significant difference. C-reactive protein (CRP) levels at birth were not significantly different between the two groups (*p* = 0.138). However, CRP levels at three days were significantly higher in the VPI group (11.0 ± 1.3 mg/dL) compared to the EPI group (7.2 ± 3.2 mg/dL), with a *p*-value less than 0.001. Lactate Dehydrogenase (LDH) levels, both at birth and at three days, were significantly higher in the EPI group compared to the VPI group. The mean LDH level at birth was 851.8 ± 72.2 UI/L in the EPI group and 468.9 ± 108.2 UI/L in the VPI group, with a *p*-value less than 0.001. Similarly, LDH levels at three days were significantly higher in the EPI group (962.3 ± 69.9 UI/L) compared to the VPI group (565.9 ± 119.0 UI/L), with a *p*-value less than 0.001.

As presented in Table 2, leukocyte counts at birth and three days were higher in the EPI group compared to the VPI group, but the difference was only statistically significant at birth (*p* = 0.036). At three days, the difference was not statistically significant (*p* = 0.052). The percentage of Polymorpho-nuclear leukocytes (PMNs) at birth was significantly higher in the VPI group (47.2 ± 20.9%) compared to the EPI group (33.4 ± 16.5%), with a *p*-value of 0.043. A similar trend was observed for PMNs at three days, with the VPI group having a higher percentage (49.6 ± 19.3%) than the EPI group (36.0 ± 14.6%), and the *p*-value was 0.030.

Table 3 presents the prevalence of abnormal laboratory findings in the study groups: Extremely Premature Infants (EPI) and Very Premature Infants (VPI). Regarding Interleukin 6 (IL-6) levels, all the EPIs (100%) had pathological levels, compared to 77.8% of the VPIs. However, the difference between the two groups was not statistically significant (*p* = 0.073). As for C-reactive protein (CRP) levels, the prevalence of pathological findings was similar in both groups, with 83.3% of EPIs and 86.1% of VPIs having abnormal levels. The difference was not statistically significant (*p* = 0.813).

Lactate Dehydrogenase (LDH) levels were also pathologically high in both groups, with 91.7% of EPIs and 83.3% of VPIs showing abnormal levels. Again, this difference was not statistically significant (*p* = 0.478). Regarding leukocyte counts, 66.7% of EPIs and 52.8% of VPIs had abnormal counts. The difference between the two groups was not statistically significant (*p* = 0.401). Lastly, the polymorphonuclear leukocytes (PMN%) proportion was pathologically high in 75.0% of EPIs and 55.6% of VPIs. The difference between the two groups was not statistically significant (*p* = 0.232).

Table 4 presents the changes in biochemical findings from birth to 3 days after birth for Extremely Premature Infants (EPI) and Very Premature Infants (VPI). In the EPI group, C-reactive protein (CRP) levels increased slightly from 6.0 ± 1.8 mg/dL at birth to 7.2 ± 3.2 mg/dL at three days, but this change was not statistically significant (*p* = 0.315). In contrast, in the VPI group, CRP levels increased significantly from 4.6 ± 2.6 mg/dL at birth to 11.0 ± 1.3 mg/dL at three days (*p* < 0.001). Lactate Dehydrogenase (LDH) levels showed a significant increase in both groups. In the EPI group, LDH levels rose from 851.8 ± 72.2 UI/L at birth to 962.3 ± 69.9 UI/L at three days (*p* = 0.003). Similarly, in the VPI group, LDH levels increased from 468.9 ± 108.2 UI/L at birth to 565.9 ± 119.0 UI/L at three days (*p* = 0.010).

Leukocyte counts decreased from birth to 3 days in both groups, but the decrease was not statistically significant. In the EPI group, leukocyte counts decreased from 15,614.0 ± 6834.4 at birth to 13,927.2 ± 5527.3 at three days (*p* = 0.513). In the VPI group, leukocyte counts decreased from 12,407.7 ± 3375.1 at birth to 10,924.0 ± 4164.5 at three days (*p* = 0.101). The percentage of Polymorphonuclear leukocytes (PMN%) slightly increased in both groups, but the change was not statistically significant. In the EPI group, PMN% increased from 33.4 ± 16.5% at birth to 36.0 ± 14.6% at three days (*p* = 0.686). In the VPI group, PMN% increased from 47.2 ± 20.9% at birth to 49.6 ± 19.3% at three days (*p* = 0.614).

Table 5 compares the assessments of umbilical cord (UC) inflammation and the severity of Retinopathy of Prematurity (ROP) between Extremely Premature Infants (EPI) and Very Premature Infants (VPI). Regarding umbilical cord inflammation, half of the EPI (50.0%) had a score of S0, indicating no inflammation, compared to 47.2% of the VPI. The EPI group had no inflammation at the S1 and S2 levels. However, 25.0% of EPI had severe inflammation (S3 and S4), whereas, in the VPI group, these severe inflammation scores were less common, with 13.9% at S3 and 11.1% at S4. Despite these differences, the overall distribution of UC inflammation levels between EPI and VPI was not statistically significant (*p* = 0.194).

Regarding the severity of ROP, none of the EPI had an ROP severity score of S0 or S1, but half had moderate to severe ROP (S2, S3, and S4). In contrast, a large portion of the VPI group (61.1%) had an ROP severity score of S0 or S1, indicating no or minimal ROP. Only a small number of VPI had moderate to severe ROP. The distribution of ROP severity was significantly different between the EPI and VPI groups (*p* = 0.002). The Spearman’s correlation analysis between UC inflammation and ROP severity indicated a statistically significant positive association (rho = 0.631, *p* < 0.001).

Table 6 compares the neonatal management strategies and patient outcomes between Extremely Premature Infants (EPI) and Very Premature Infants (VPI). Regarding oxygen supplementation, all EPIs (100%) required this intervention, as did most of the VPIs (94.4%). The difference between groups was not statistically significant (*p* = 0.404). However, the duration of oxygen supplementation was significantly longer in the EPI group, with a mean of 37.2 ± 11.6 days compared to 21.8 ± 12.4 days in the VPI group (*p* = 0.004). For mechanical ventilation, the percentage of patients requiring this intervention was not significantly different between EPI and VPI (*p* = 0.861), nor was the duration of ventilation (*p* = 0.503). 

Continuous Positive Airway Pressure (CPAP) was used in 50.0% of EPI and 77.8% of VPI, a difference that approached but did not reach statistical significance (*p* = 0.066). The duration of CPAP use was not significantly different between the two groups (*p* = 0.741). Surfactant supplementation was administered in 33.3% of EPI and 52.8% of VPI, with no statistically significant difference (*p* = 0.242). However, erythrocyte concentrate administration was significantly more common in the VPI group (88.9%) compared to the EPI group (41.7%) (*p* = 0.001). The EPI group received a significantly higher number of erythrocyte concentrate packs (mean 3.0 ± 1.6) than the VPI group (mean 2.0 ± 1.3) (*p* = 0.034). Mortality was significantly higher in the EPI group (41.7%) compared to the VPI group (5.6%) (*p* = 0.002).

Table 7 presents the results of a multivariate regression analysis identifying risk factors for the development of Retinopathy of Prematurity (ROP) stage 2 or above in Extremely Premature Infants (EPI) and Very Premature Infants (VPI). Regarding clinical parameters, the duration of oxygen supplementation greater than 18 days was significantly associated with an increased risk of developing ROP stage 2 or above (OR = 2.48, 95% CI 1.31–6.14, *p* = 0.009). Similarly, the duration of mechanical ventilation over ten days also presented a significant risk (OR = 1.95, 95% CI 1.16–4.70, *p* = 0.036). Spending over ten days on Continuous Positive Airway Pressure (CPAP) was also significantly associated with an increased risk (OR = 1.33, 95% CI 1.01–4.96, *p* = 0.045).

**Figure 1:**
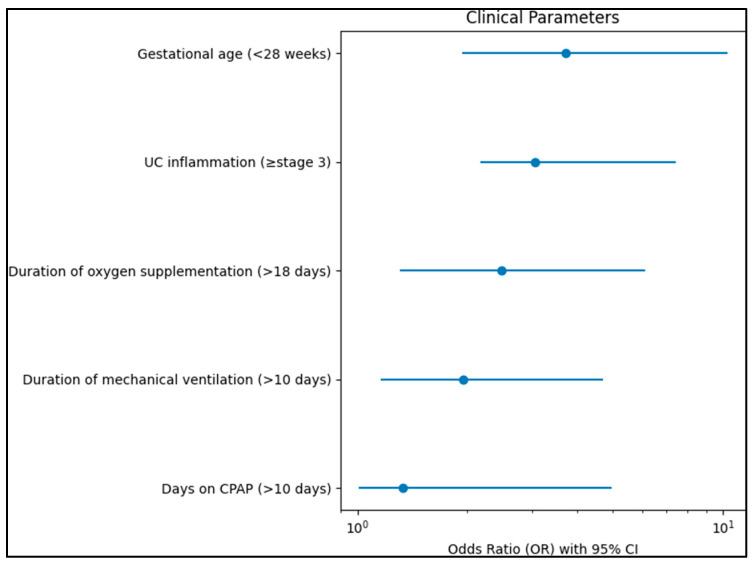
Clinical parameters identified as risk factors for ROP stage 2 or above.

**Figure 2:**
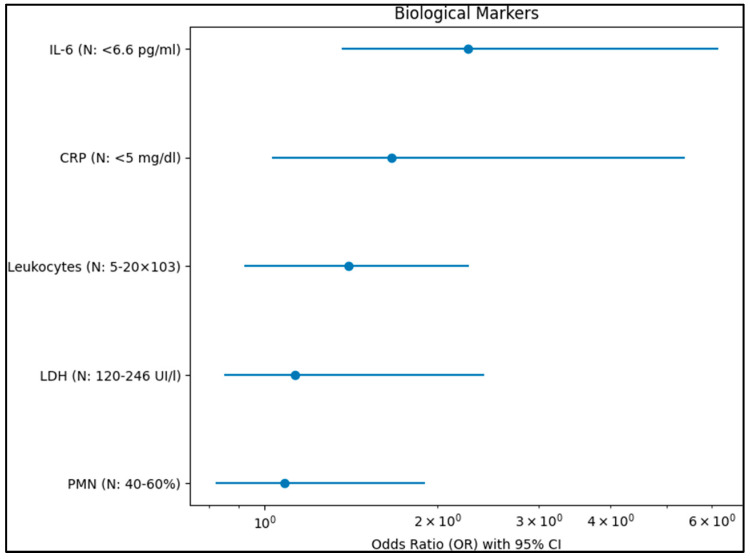
Biological markers identified as risk factors for ROP stage 2 or above.

Gestational age of less than 28 weeks was a significant risk factor (OR = 3.72, 95% CI 1.93–10.28, *p* < 0.001), as presented in Figure 1. Additionally, umbilical cord inflammation at or above stage 3 was significantly associated with an increased risk of ROP stage 2 or above (OR = 3.06, 95% CI 2.17–7.42, *p* = 0.001). Regarding biological markers outside the normal range, elevated levels of C-reactive protein (CRP) (OR = 1.66, 95% CI 1.03–5.39, *p* = 0.001) and Interleukin-6 (IL-6) (OR = 2.26, 95% CI 1.36–6.15, *p* < 0.001) were both significantly associated with an increased risk of developing ROP stage 2 or above. The levels of lactate dehydrogenase (LDH), leukocytes, and polymorpho-nuclear leukocytes (PMN) were not significantly associated with the risk of developing ROP stage 2 or above (*p* = 0.194, *p* = 0.240, and *p* = 0.275, respectively), as described in Figure 2.

## 4. Discussion

### 4.1. Literature Findings

The present study aimed to elucidate the relationship between the inflammatory status of extremely premature (EPI) and very premature infants (VPI) and the development and progression of retinopathy of prematurity (ROP), a significant health concern in these vulnerable groups. Our results shed light on various aspects of this complex relationship, contributing to the existing body of knowledge on the role of inflammation in ROP’s pathogenesis. It was found that EPIs had significantly higher levels of Interleukin-6 (IL-6) and Lactate Dehydrogenase (LDH) compared to VPIs, suggesting a more pronounced inflammatory response in this group. Additionally, the EPI group showed higher leukocyte counts at birth, indicating a heightened immune response. These findings align with our first hypothesis that higher levels of inflammatory markers are associated with an increased risk of ROP development and progression. However, at three days, C-reactive protein (CRP) levels were significantly higher in the VPI group (11.0 mg/dL vs. 7.2 mg/dL), indicating a delayed inflammatory response.

While we observed significantly higher CRP levels at three days in the VPI group, suggestive of a delayed inflammatory response, it’s important to consider the initially high leukocyte counts at birth in this group. This initial high count indicates that these infants already had systemic inflammation at birth, challenging our initial interpretation of a delayed response. Thus, the term “delayed” might not accurately reflect the dynamics of the inflammatory process in the VPI group. Further investigation is required to fully understand the complexities of the inflammatory response in these patients. We postulate that external or environmental factors, such as oxygen supplementation and mechanical ventilation, might have exacerbated the systemic inflammation in the VPI group. Additionally, other unknown factors, which could be related to the individual health status of the infants or varying medical interventions, may also contribute to systemic inflammation. It’s important to highlight that our study did not specifically investigate these potential triggers; hence, our understanding of the exact causes remains limited.

Interestingly, while EPIs showed a distinct inflammatory profile, the prevalence of pathological findings in IL-6, CRP, LDH, Leukocytes, and PMNs was not significantly different between the EPI and VPI groups. This suggests that the risk of developing ROP is not solely dependent on the presence of abnormal inflammatory markers but may also be influenced by other factors such as gestational age, birth weight, and medical interventions. Regarding ROP severity, EPIs were more likely to have moderate to severe ROP, a statistically significant finding. Furthermore, we identified a significant positive correlation between umbilical cord inflammation and ROP severity, supporting the role of inflammation in the pathogenesis of ROP.

The multivariate regression analysis identified several risk factors for developing ROP stage 2 or above. These included longer durations of oxygen supplementation, mechanical ventilation, and CPAP use, gestational age less than 28 weeks, and umbilical cord inflammation at or above stage 3. Elevated levels of CRP and IL-6 were also significantly associated with an increased risk of developing ROP stage 2 or above. Thus, our study reaffirms the role of inflammation in the pathogenesis of ROP, with distinct inflammatory profiles associated with varying degrees of ROP severity, as previously hypothesized and described [32,33]. Furthermore, we identified several risk factors for ROP development, including gestational age, duration of oxygen supplementation, mechanical ventilation, CPAP use, and elevated levels of specific inflammatory markers. These findings aid in the early identification and management of infants at risk for developing ROP, potentially improving outcomes in this vulnerable population. However, further research is needed to fully elucidate the complex interplay between inflammation and ROP development and progression.

Another study confirmed the link between surfactant treatment, identified in previous research as a significant risk factor for Retinopathy of Prematurity (ROP) [34], and the incidence of ROP in neonates diagnosed with moderate to severe respiratory distress syndrome (RDS) shortly after birth. In comparison, a unique aspect of our study was that surfactant treatment was administered strictly as a therapeutic intervention, not prophylactically, as in most studies. This contrasts with the typical approach where most or all infants in a study receive preventative surfactant treatment. Nevertheless, as the most recent guidelines suggest for treating ROP, dexamethasone eye drops are safe to use and recommended for extremely premature and very premature newborns with ROP [35].

A considerable body of research has been dedicated to the incidence of ROP in recent years. Yet, there remains to be a unified agreement concerning the most critical predictors of ROP, its various stages, or the precise mechanisms through which these risk factors impact ROP onset and severity [35]. Existing knowledge points to several well-recognized risk factors, such as gestational age (GA) and birth weight (BW) [36,37], oxygen and surfactant administration [38], mechanical ventilation [39,40], and blood transfusions [41] that were confirmed in our study as significant risk factors. More recent research has highlighted additional factors strongly linked to ROP, including slow weight gain [42], bronchopulmonary dysplasia [43], and the use of inhaled nitric oxide [44].

Intriguingly, certain perinatal characteristics, such as intrauterine hypoxia, necrotizing enterocolitis, and hemolytic disease, have been reported to have a reverse association with ROP [39]. Other research has posited that BW is the most reliable predictor of ROP (any stage) in a multivariate model [41], outperforming ventilation as a statistically significant risk factor [44]. In contrast to the majority of studies in the literature [40,41,42,43,44,45], our findings did not unveil any significant association between GA and ROP when examined in a multivariate model, except for those born before 28 weeks of gestation and the development of ROP stage 2 or higher. Although GA was inversely related to ROP in the univariate analysis, its statistical significance diminished when other factors were incorporated into the model, indicating that BW and ventilation were superior predictors in our multivariate model. These findings underscore the complex interplay of factors contributing to ROP and the need for further research to better understand their individual and combined effects. This will be instrumental in improving prediction, prevention, and treatment strategies for ROP in extremely premature and very premature neonates.

The observed discrepancy in our findings, compared to some other studies, could be attributed to our study’s exclusive focus on extremely and very preterm infants. Other studies have included infants with a gestational age (GA) of 32 weeks or more [46,47] or have not delineated an upper GA limit, which could encompass infants with a GA of 32 weeks or more [42,48]. As a result, the contrast between groups of high and low GA regarding the incidence of Retinopathy of Prematurity (ROP) may be more pronounced, and the relationship between GA and ROP could be emphasized more effectively in these studies.

The use of continuous positive airway pressure (CPAP) treatment has been demonstrated to correlate significantly with ROP (any stage) in multivariate models [41,49]. Similarly, in our research, it was displayed as a significant risk factor with ROP stage 2 or higher in multivariate analysis. The more perinatal factors we consider, the greater our ability to identify ROP risk factors, understand their correlations with ROP, and evaluate the statistical significance of these associations. This understanding is primarily due to the interconnected mechanisms among risk factors. Any newly identified risk factor enhances the statistical model, potentially aiding ophthalmologists in diagnosing, predicting, and treating ROP [50]. Hence, to understand better the intricate interactions among various perinatal characteristics, more research is needed to determine how these factors affect ROP onset and progression and to refine clinical practice guidelines for ROP.

To the best of our understanding of the current research, LDH and CRP levels have been infrequently examined as potential ROP risk factors. At the same time, our study identified a substantial association between these CRP and IL-6 and ROP (stage 2 or higher) without proving LDH as a significant risk factor. Regarding the inflammatory markers selected for this study, IL-6 was chosen for its well-established role as a primary pro-inflammatory cytokine and its recognized association with neonatal complications, such as sepsis, necrotizing enterocolitis, and even ROP [9,51]. Similarly, CRP, an acute-phase reactant that rises rapidly during inflammation and infection, was selected for its routine use in monitoring these conditions and its proposed relationship with ROP development [17,51]. The combined assessment of these two markers allows for a more comprehensive understanding of the neonates’ inflammatory status. Nevertheless, a wider panel of inflammatory markers should be studied in future studies that could potentially be more reliable in the study of ROP.

### 4.2. Study Limitations

The study has several limitations that should be acknowledged. The first limitation is related to the sample size and sampling method. With a sample size of 48 neonates, the study may have needed more statistical power to detect significant associations between inflammatory markers and ROP, especially if the effect sizes were small. Additionally, the convenience sampling method might have led to selection bias, as it may have yet to fully represent the population of extremely premature and very premature neonates. It would have been ideal to perform a random sampling to ensure a more representative population sample, reducing the risk of bias.

The second limitation is the study’s exclusion of neonates delivered via Cesarean section, neonates with a gestational age of 22 weeks or less, and neonates with congenital anomalies or critical illnesses. While these exclusions were made to maintain a homogeneous study population, they limit the generalizability of the findings to all premature neonates. Moreover, excluding neonates who did not survive the neonatal period might have led to an underestimation of the association between inflammatory markers and ROP, as these neonates might have had higher levels of inflammation and a higher risk of ROP. Therefore, the findings of this study should be interpreted with caution and may not apply to all populations of premature neonates. Future research should include a more diverse population of neonates and possibly a larger sample size to validate and expand upon these findings. 

## 5. Conclusions

The findings of this study underline the critical role inflammation plays in the development and progression of Retinopathy of Prematurity (ROP) in extremely premature and very premature neonates. Our data confirm the initial hypotheses that higher levels of inflammatory markers are associated with an increased risk of ROP development and progression and that neonates with ROP exhibit a distinct inflammatory profile compared to those without. The significant disparities in birth weight, IL-6 levels, CRP levels, and LDH levels between extremely and very premature infants underscore the effect of gestational age and birth weight on inflammatory status. Furthermore, our findings revealed that the duration of oxygen supplementation, mechanical ventilation, prolonged CPAP use, gestational age of fewer than 28 weeks, and umbilical cord inflammation at or above stage 3 were significant risk factors for the development of ROP stage 2 or above. Elevated levels of CRP and IL-6 were also significantly associated with an increased risk of developing ROP stage 2 or above, highlighting their potential as biomarkers for ROP risk prediction. Overall, this study underscores the crucial need for early and consistent monitoring of inflammatory markers in premature neonates and points towards the potential of inflammation-targeted therapeutic strategies in mitigating the risk and severity of ROP in this vulnerable population. Future research should focus on validating these findings in larger, multi-center cohorts and exploring the mechanistic pathways linking inflammation and ROP to refine our understanding further and enable the development of targeted interventions.

## Figures and Tables

**Table 1:** Demographics and clinical characteristics.

Variables	Total (*n =* 48)	EPI (*n =* 12)	VPI (*n =* 36)	*p*
Weight, grams (mean ± SD)	1291.8 ± 405.8	864.5 ± 231.4	1392.7 ± 364.2	<0.001 *
Weight range				0.004
500–1000 g	15 (31.3%)	8 (66.7%)	7 (19.4%)	
1000–1500 g	20 (41.6%)	4 (33.3%)	16 (44.4%)	
1500–2000 g	13 (27.1%)	0 (0.0%)	13 (36.2%)	
Gender				0.738
Male	26 (54.2%)	6 (50.0%)	20 (55.6%)	
Female	22 (45.8%)	6 (50.0%)	16 (44.4%)	
Gestational age (weeks)				–
24–25	4 (8.3%)	4 (33.3%)	0 (0.0%)	
26–27	8 (16.7%)	8 (66.7%)	0 (0.0%)	
28–29	7 (14.6%)	0 (0.0%)	7 (19.4%)	
30–31	29 (60.4%)	0 (0.0%)	29 (80.6%)	

Data described as *n* (%) and compared using the Chi-square test and Fisher’s exact unless specified differently; EPI—Extremely Premature Infants (<28 weeks of gestation); VPI—Very Premature Infants (28–32 weeks of gestation); SD—Standard Deviation; * Student’s *t*-test (unpaired).

**Table 2:** Comparison of inflammatory markers by the level of prematurity.

Variables	Total (*n =* 48)	EPI (*n =* 12)	VPI (*n =* 36)	*p*
IL-6 (*N*: <6.6 pg/mL)	319.1 ± 78.6	638.2 ± 122.7	151.1 ± 26.7	<0.001
CRP at birth (*N*: <5 mg/dL)	5.5 ± 1.1	6.0 ± 1.8	4.6 ± 2.6	0.138
CRP at 3 days (*N*: <5 mg/dL)	10.0 ± 0.9	7.2 ± 3.2	11.0 ± 1.3	<0.001
LDH at birth (*N*: 120–246 UI/L)	596.5 ± 46.3	851.8 ± 72.2	468.9 ± 108.2	<0.001
LDH at 3 days (*N*: 120–246 UI/L)	698.0 ± 45.6	962.3 ± 69.9	565.9 ± 119.0	<0.001
Leukocytes at birth (*N*: 5–20 × 10^3^)	13,618.3 ± 5073.9	15,614.0 ± 6834.4	12,407.7 ± 3375.1	0.036
Leukocytes at 3 days (*N*: 5–20 × 10^3^)	11,072.9 ± 5414.6	13,927.2 ± 5527.3	10,924.0 ± 4164.5	0.052
PMNs at birth (*N*: 40–60%)	39.8 ± 18.7	33.4 ± 16.5	47.2 ± 20.9	0.043
PMNs at 3 days (*N*: 40–60%)	42.5 ± 17.0	36.0 ± 14.6	49.6 ± 19.3	0.030

*N*—Normal range; Data described as mean ± SD and analyzed using Student’s *t*-test; EPI—Extreme prematurity (<28 weeks of gestation); VPI—Very Preterm (28–32 weeks of gestation); SD—Standard Deviation; IL-6—Interleukin 6; CRP—C-reactive protein; LDH—Lactate Dehydrogenase; PMN—Polymorphonuclear leukocytes.

**Table 3:** Prevalence of abnormal laboratory findings in the EPI and VPI study groups.

Variables	Total (*n =* 48)	EPI (*n =* 12)	VPI (*n =* 36)	*p*
IL-6 (*N*: <6.6 pg/mL)				0.073
Pathological	40 (83.3%)	12 (100%)	28 (77.8%)	
Normal	8 (16.7%)	0 (0.0%)	8 (22.2%)	
CRP (*N*: <5 mg/dL)				0.813
Pathological	41 (85.4%)	10 (83.3%)	31 (86.1%)	
Normal	7 (14.6%)	2 (16.7%)	5 (13.9%)	
LDH (*N*: 120–246 UI/L)				0.478
Pathological	41 (85.4%)	11 (91.7%)	30 (83.3%)	
Normal	7 (14.6%)	1 (8.3%)	6 (16.7%)	
Leukocytes (*N*: 5–20 × 10^3^)				0.401
Pathological	27 (56.3%)	8 (66.7%)	19 (52.8%)	
Normal	21 (43.8%)	4 (33.3%)	17 (47.2%)	
PMN% (*N*: 40–60%)				0.232
Pathological	29 (60.4%)	9 (75.0%)	20 (55.6%)	
Normal	19 (39.6%)	3 (25.0%)	16 (44.4%)	

*N*—Normal range; Data described as *n* (%) and calculated using the Chi-square test and Fisher’s exact unless specified differently; EPI—Extreme prematurity (<28 weeks of gestation); VPI—Very Preterm (28–32 weeks of gestation); SD—Standard Deviation; IL-6—Interleukin 6; CRP—C-reactive protein; LDH—Lactate Dehydrogenase; PMN—Polymorphonuclear leukocytes.

**Table 4:** Biochemical findings at birth and three days after birth.

	EPI (*n =* 12)			VPI (*n =* 36)		
Biological Parameters	At Birth	At 3 Days	*p*-Value	At Birth	At 3 Days	*p*
CRP (*N*: <5 mg/dL)	6.0 ± 1.8	7.2 ± 3.2	0.315	4.6 ± 2.6	11.0 ± 1.3	<0.001
LDH (*N*: 120–246 IU/L)	851.8 ± 72.2	962.3 ± 69.9	0.003	468.9 ± 108.2	565.9 ± 119.0	0.01
Leukocytes (*N*: 5–20 × 10^3^)	15,614.0 ± 6834.4	13,927.2 ± 5527.3	0.513	12,407.7 ± 3375.1	10,924.0 ± 4164.5	0.101
PMN (*N*: 40–60%)	33.4 ± 16.5	36.0 ± 14.6	0.686	47.2 ± 20.9	49.6 ± 19.3	0.614

*N*—Normal range; Data described as mean ± SD and analyzed using Student’s *t*-test (paired); EPI—Extreme prematurity (<28 weeks of gestation); VPI—Very Preterm (28–32 weeks of gestation); SD—Standard Deviation; CRP—C-reactive protein; LDH—Lactate Dehydrogenase; PMN—Polymorphonuclear leukocytes; IU—International units.

**Table 5:** The assessment of umbilical cord inflammation and ROP severity between EPI and VPI neonates.

Variables	EPI (*n =* 12)	VPI (*n =* 36)	*p*
UC inflammation			0.194
S0	6 (50.0%)	17 (47.2%)	
S1	0 (0.0%)	6 (16.7%)	
S2	0 (0.0%)	5 (13.9%)	
S3	3 (25.0%)	4 (13.9%)	
S4	3 (25.0%)	4 (11.1%)	
ROP severity			0.002
S0	0 (0.0%)	22 (61.1%)	
S1	0 (0.0%)	3 (8.3%)	
S2	6 (50.0%)	7 (19.4%)	
S3	4 (33.3%)	3 (8.3%)	
S4	2 (16.7%)	1 (2.8%)	

Data described as *n* (%) and calculated using the Chi-square test and Fisher’s exact unless specified differently; UC—Umbilical Cord; ROP—Retinopathy of prematurity; EPI—Extreme prematurity (<28 weeks of gestation); VPI—Very Preterm (28–32 weeks of gestation).

**Table 6:** Neonatal management and patient outcomes.

Variables	EPI (*n =* 12)	VPI (*n =* 36)	*p*
Oxygen supplementation (*n*, %)	12 (100%)	34 (94.4%)	0.404
Days of oxygen supplementation (mean ± SD)	37.2 ± 11.6	21.8 ± 12.4	0.004
Mechanical ventilation (*n*, %)	4 (33.3%)	13 (36.1%)	0.861
Days of mechanical ventilation (mean ± SD)	10.5 ± 8.1	12.3 ± 7.9	0.503
CPAP (*n*, %)	6 (50.0%)	28 (77.8%)	0.066
Days spent on CPAP (mean ± SD)	12.0 ± 9.4	11.3 ± 5.0	0.741
Surfactant supplementation (*n*, %)	4 (33.3%)	19 (52.8%)	0.242
Erythrocyte concentrate administration (*n*, %)	5 (41.7%)	32 (88.9%)	0.001
Erythrocyte concentrate (number of packs)	3.0 ± 1.6	2.0 ± 1.3	0.034
Mortality (*n*, %)	5 (41.7%)	2 (5.6%)	0.002

Data described as *n* (%) and calculated using the Chi-square test and Fisher’s exact unless specified differently; EPI—Extreme prematurity (<28 weeks of gestation); VPI—Very Preterm (28–32 weeks of gestation); CPAP—Continuous positive airway pressure; SD—Standard deviation.

**Table 7:** Multivariate regression analysis for ROP stage 2 or above risk factors.

Variables	OR	95% CI	*p*
Clinical parameters			
Duration of oxygen supplementation (>18 days)	2.48	1.31–6.14	0.009
Duration of mechanical ventilation (>10 days)	1.95	1.16–4.70	0.036
Days on CPAP (>10 days)	1.33	1.01–4.96	0.045
Gestational age (<28 weeks)	3.72	1.93–10.28	<0.001
UC inflammation (≥stage 3)	3.06	2.17–7.42	0.001
Biological markers (outside the normal range)			
CRP (*N*: <5 mg/dL)	1.66	1.03–5.39	0.001
LDH (*N*: 120–246 IU/L)	1.13	0.85–2.41	0.194
Leukocytes (*N*: 5–20 × 10^3^)	1.40	0.92–2.27	0.240
PMN (*N*: 40–60%)	1.08	0.82–1.90	0.275
IL-6 (*N*: <6.6 pg/mL)	2.26	1.36–6.15	<0.001

*N*—Normal range; OR—Odds ratio; CI—Confidence interval; CPAP—Continuous positive airway pressure; UC—Umbilical Cord; CRP—C-reactive protein; LDH—Lactate Dehydrogenase; PMN—Polymorphonuclear leukocytes; IL—Interleukins; IU—International units.

## Data Availability

Data is available upon request.
